# The Application of Chemical Polishing in TEM Sample Preparation of Zirconium Alloys

**DOI:** 10.3390/ma13051036

**Published:** 2020-02-25

**Authors:** Fusheng Li, Shilei Li, Huan Tong, Hainan Xu, Yanli Wang

**Affiliations:** State Key Laboratory for Advanced Metals and Materials, University of Science and Technology Beijing, Beijing 100083, China; 18810562702@163.com (F.L.); lishilei@ustb.edu.cn (S.L.); tonghuan416@163.com (H.T.);

**Keywords:** zirconium alloys, hydride, bend contour, TEM

## Abstract

Hydride artefacts are commonly induced by the TEM sample preparation process in Zirconium alloys as hydrogen-sensitive metals, including electron polishing and focused ion beam (FIB) technology. In the research, we present the application of chemical polishing with a solution of 10HF:45HNO_3_:45H_2_O to prepare the disk samples for TEM observation in zirconium alloys. The thinning efficiency of chemical polishing is 25 μm per minute. XRD patterns indicate that the chemical polishing actually eliminates the macro- and micro-stress induced by mechanical grinding. TEM observation demonstrates that chemical polishing reduces the amount of hydride artefacts, especially hydrides with large size. It is proposed that induced stress provides driving force for hydride artefact formation. Compared with traditional mechanical grinding, the advantages of chemical polishing are high efficiency, free of induced stress, less induced hydride artefacts and bend contours.

## 1. Introduction

Zirconium alloys have been widely used as fuel cladding materials in light water and pressurized water reactors due to their low thermal neutron cross section, good corrosion resistance and mechanical properties [1,2,3]. These zirconium claddings experience microstructure evolution and mechanical property changes under fast neutron irradiation and corrosion reaction [4]. Transmission electron microscopy (TEM) is an important tool for microstructure observation in zirconium alloys research. However, many challenges actually present in the TEM sample preparation process of zirconium alloys. The induced hydride artefacts are the major challenge during TEM sample preparation, including traditional twin-jet electron polishing and focused ion beam (FIB) milling. With regard to FIB milling to minimize hydride artefacts in Zirconium alloys, the methods suggested are gradient ion milling and cryo-FIB [5,6]. Obvious defects are present in the two suggested methods. The observable region with a width about 2 μm is indeed small in the TEM sample prepared by gradient ion milling [5]. Actually, the hydride artefacts in TEM samples prepared by cryo-FIB could be reduced, not eliminated [6]. Based on this, traditional twin-jet electron polishing to prepare TEM sample of Zirconium alloys requires more attention to reduce the hydride artefacts. 

For twin-jet electron polishing, a disk sample with 3 mm in diameter and 50 μm in thickness is required. Traditionally, the sample with a thickness of 500 μm after wire-cut electrical discharge machining (WEDM) was mechanically grinded with sandpaper to about 100 μm in thickness. Then, the thin lamella was punched into a disk with 3 mm in diameter. Finally, these disk samples were mechanically grinded again to 50 μm for twin-jet electron polishing. The TEM sample preparation by traditional mechanical grinding is really time-consuming and excruciating when numerous TEM samples were required to prepare. In addition, it is worth noting that there would be mechanical stress induced by grinding [7,8]. The stress induced by mechanical grinding would have a strong impact on the TEM observation when the sample is extremely thin, less than 100 nm. It is a well-known fact that the hydrogen would redistribute as a consequence of stress gradients resulting in formation of zirconium hydride [5,9,10,11,12,13,14]. Obviously, efforts to reduce stress in zirconium TEM samples should be taken when twin-jet electron polishing was required for sample preparation. Chemical polishing represents a convenient approach to thin-foil preparations for a wide range of metals and alloys [15]. The materials could be thinned uniformly through the reaction of solution and metals with the appropriate solution. Related to zirconium alloys, chemical polishing is generally used in electron backscattered diffraction (EBSD) sample preparation to remove scratches induced by mechanical grinding. A solution of 10HF:45HNO_3_:45H_2_O for chemical polishing was usually used [2,16]. Based on this, we consider whether the chemical polishing could be applied to the preparation of disk samples for TEM. If available, plenty of disk samples for twin-jet electron polishing could be prepared within a short time, meanwhile avoiding the induced stress by mechanical grinding.

In this research, we present the application of chemical polishing in preparing zirconium disk samples for twin-jet electron polishing with a slight mechanical grinding. The advantage of chemical polishing compared with traditional mechanical grinding in preparing zirconium disk samples for TEM observation is high efficiency (plenty of disk samples can be prepared within an hour) and none stress induced. The application of chemical polishing in preparing disk samples for TEM observation will contribute to the development of research related to zirconium alloys.

## 2. Materials and Methods

The materials investigated in this study were Zircaloy-4 sheets with a chemical composition of 1.3 wt.%Sn, 0.2 wt.%Fe, 0.1 wt.%Cr, 0.1 wt.%O and 98.3 wt.%Zr. The microstructure of Zircaloy-4 is equiaxed grain and the average grain size is 8 μm.

**Figure 1:**
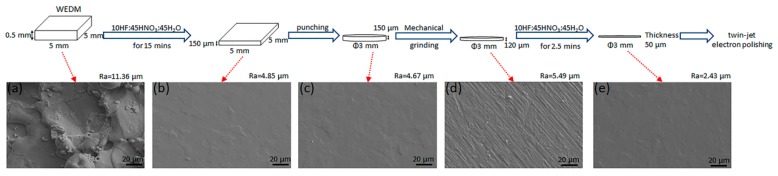
The schematic diagram of TEM sample preparation process by chemical polishing in Zirclaoy-4. SEM images of surface morphology taken from every step are shown in (**a**–**e**), respectively.

For transmission electron microscope (TEM, FEI Tecnai G2 F30, FEI, MA, USA) sample preparation, Zircaloy-4 sheets were firstly machined to a size of 5 mm × 5 mm × 0.5 mm by wire-cut electrical discharge machining (WEDM, DK7725, NC equipment factory of Ningbo Jiangbei, Jiangbei, China). Then, two different processes were performed to obtain disk specimens of 3 mm in diameter for the next twin-jet electron polishing: traditional mechanical grinding and chemical polishing. For traditional mechanical grinding, Zircaloy-4 specimens with a thickness of 500 μm were grinded with 2000# sandpaper to about 100 μm. Then, a disk specimen of 3 mm in diameter was punched. At last, the 3 mm disk specimen was grinded again to about 50 μm in thickness. The 3 mm disk specimen with a thickness of 50 μm was prepared for twin-jet electron polishing to obtain the final TEM sample. The schematic diagrams of preparation process of TEM sample by chemical polishing are shown in Figure 1. The samples of 500 μm in thickness after WEDM were chemically polished with a solution of 10HF:45HNO_3_:45H_2_O for 15 min. The thickness of polished sample was about 150 μm, which met thickness requirements of next punching. Then the 3 mm disk specimen was punched. A slight mechanical grinding was applied to both sides of the disk. After grinding, the thickness of the disk was about 120 μm. Another chemical polishing was performed to reduce the thickness of disk to 50 μm. At last, the disk was prepared for the last twin-jet electron polishing. Those disk samples were polished in a solution of 10% perchloric acid and 90% ethanol with a current of 60 mA at a temperature of −40 °C For comparison, the traditional mechanical grinding and chemical polishing samples were then observed by TEM.

The roughness of sample surface was measured with OLYMPUS LEXT OLS4100 laser scanning confocal microscope (LSCM) (Tokyo, Japan). Totally 10 lines with a length of 2.5 mm in each sample were measured, the average value of which was set as the roughness of each sample. Here, the Ra value was used. A ZEISS Supra 55 scanning electron microscope (SEM) (Jena, Gremany) was used to observe the apparent condition. The observation of Zircaloy-4 samples was performed in FEI Tecnai G2 F30 field-emission TEM (MA, USA) operated at 300 kV.

## 3. Result and Discussion

### 3.1. Thinning Efficiency of Chemical Polishing

**Figure 2:**
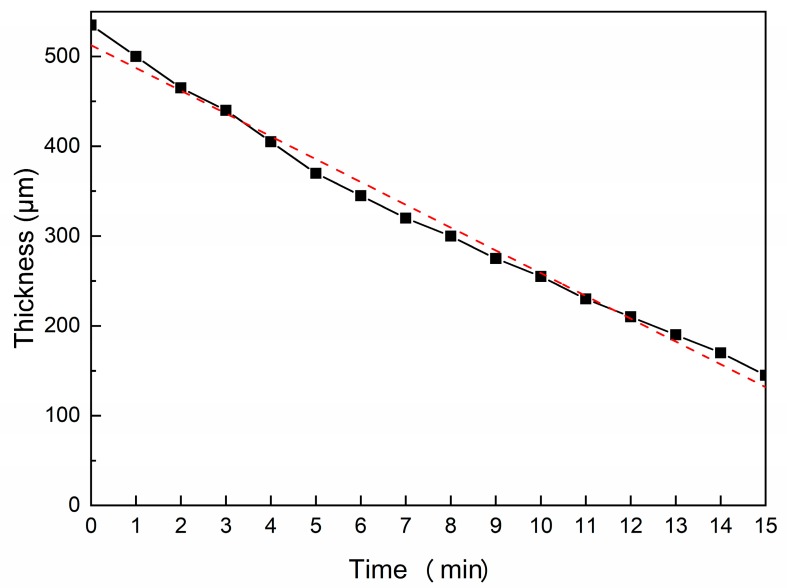
Thickness of Zircaloy-4 specimen as a function of polishing time. It is shown that the thinning is relatively homogeneous with polishing time.

The thickness of Zircaloy-4 thin-foil reduces from about 500 μm to about 150 μm after chemical polishing for 15 min. To detect the homogeneity of chemical polishing, the thickness of thin-foil was detected every minute after polishing, as shown in Figure 2. There is an obvious linear relationship between the thickness of thin-foil and polishing time. The maximum value of thickness reduction per minute is 35 μm and the minimum is 20 μm. Overall, it can be assumed that the average thickness reduction per minute is 25 μm, which could be used for estimation of chemical polishing time. It should be noted that continuous stirring is needed in chemical polishing to release the generated hydrogen gases immediately. The stirrer used should be made from plastic, not glass or metal, in hydrofluoric acid solution. After mechanical grinding, the thickness of thin-foil is about 120 μm, another chemical polishing was performed. The time of second chemical polishing is 2.5 min. The final thickness could be kept at 50 μm for twin-jet electron polishing. The thinning efficiency of chemical polishing is 25 μm per minute without connection to the thickness of thin-foil.

On the other hand, chemical polishing could also improve the surface condition. As shown in Figure 1, the average surface roughness after WEDM measured by LSCM was 11.36 μm. Rough surface topography could be observed in the specimen after WEDM shown in Figure 1a. After chemical polishing for 15 min, the surface roughness drastically reduces to 4.85 μm. As shown in Figure 1b, slight trace of zirconium grains could be observed. Then the punching was performed to obtain disk samples with 3 mm in diameter. To obtain smoother surface, a slight mechanical grinding with 2000# sandpaper was applied. Now, the surface roughness is 5.49 μm. Another chemical polishing for 2.5 min makes the surface roughness down to 2.43 μm in the end. Those disk samples were prepared for the final twin-jet electron polishing to obtain TEM samples.

### 3.2. Stress Induced by Mechanical Grinding

**Figure 3:**
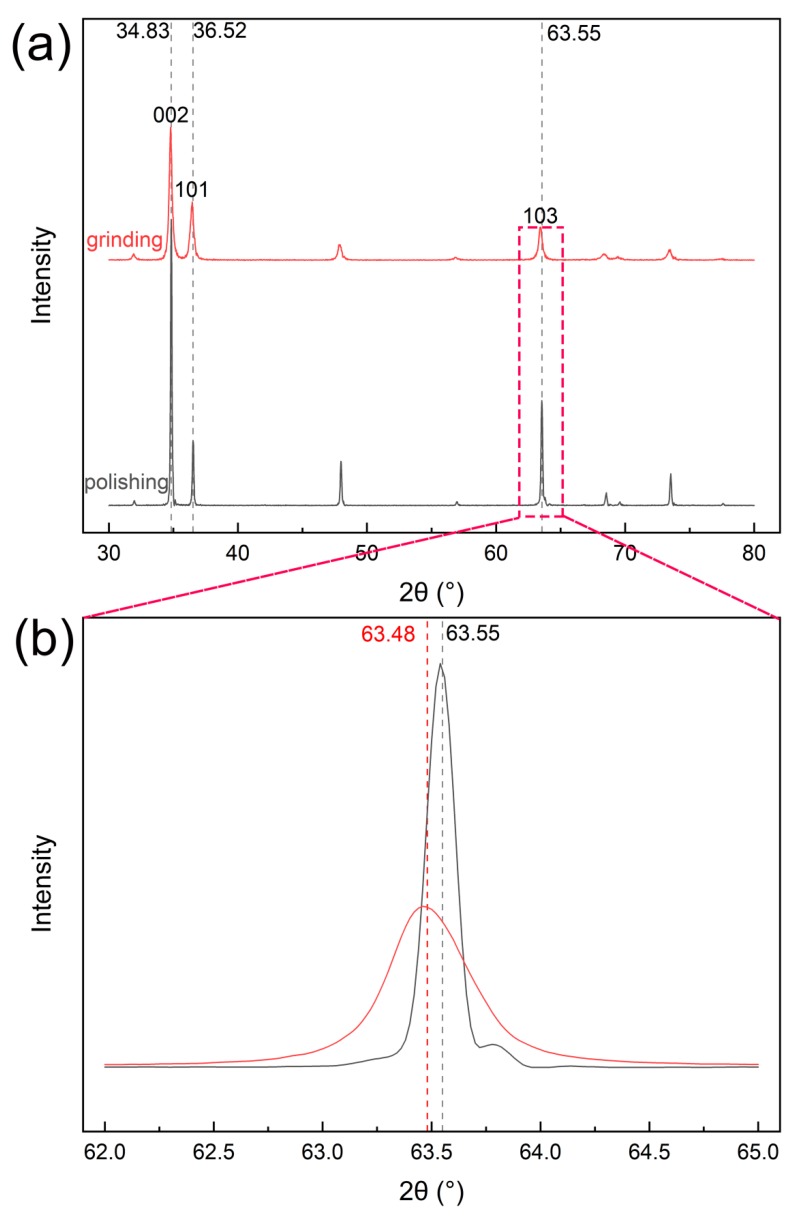
The rough scanning from 30° to 80° (**a**) and fine scanning from 62° to 65° (**b**) XRD patterns of Zircaloy-4 after mechanical grinding (red line) and chemical polishing (gray line). The grey line indicating 63.55° is standard pattern of (103) diffraction peak in α-Zr taken from PDF#65-3366.

To illustrate whether the chemical polishing would remove the stress induced by mechanical grinding, the stress of sample after mechanical grinding and chemical polishing was detected by XRD. There might be macro-stress and micro-stress induced by mechanical grinding. Shown in XRD patterns, the macro-stress leads to the excursion of diffraction peaks and the micro-stress results in the widening of diffraction peaks [17,18]. The XRD patterns of Zircaloy-4 after mechanical grinding and chemical polishing are shown in Figure 3. Here, we merely illustrate the macro- and micro-stress qualitatively. Obviously, the diffraction peaks of mechanical grinding are widening than these after chemical polishing. It could be observed distinctly in Figure 3b. The widening of diffraction peaks in grinding condition indicates that micro-stress is indeed induced by mechanical grinding. In other words, chemical polishing could remove the micro-stress induced by mechanical grinding. As for macro-stress, it is estimated according to the shift of diffraction peaks compared with normal patterns. As shown in Figure 3b, the (103) diffraction peaks after chemical polishing coincide exactly with standard patterns, indicating a stress-free condition. However, large excursion could be observed when compared the (103) diffraction peaks after mechanical grinding with standard patterns. The mechanical grinding actually induces macro-stress to the specimen and chemical polishing could remove the induced macro-stress. Totally, the chemical polishing could eliminate the stress, including macro- and micro-stress, induced by mechanical grinding.

### 3.3. Hydride Artefact

**Figure 4:**
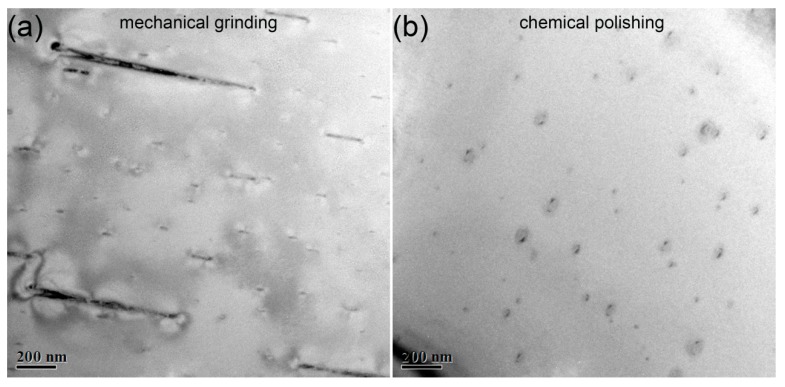
**Bright field** (BF)-TEM images of Zircaloy-4 specimens prepared by mechanical grinding (**a**) and chemical polishing (**b**), showing the microstructure of induced hydride artefacts.

One concern about sample preparation by chemical polishing for hydrogen-sensitive metals, zirconium included, is that hydride artefacts could be induced. The hydrogen ions widely distributing in the acid solution trend to react with zirconium to form various hydride artefacts [19,20]. To illustrate the effect of chemical polishing on the induced hydride artefacts, TEM observation was performed in the specimens prepared by chemical polishing and traditional mechanical grinding. The bright-field TEM images of samples prepared by mechanical grinding and mechanical polishing are shown in Figure 4a,b, respectively. Numerous tiny phases with a size below 100 nm are observed in both Figure 4a,b, which are confirmed to be δ-type hydrides. Some large δ-type hydrides with a size over 1 μm can also be observed in TEM sample prepared by mechanical grinding, however, none in sample prepared by chemical polishing. Obviously, the chemical polishing reduces the hydride artefacts, especially eliminating the large size δ-type hydride artefacts. Barrow also reported the similar structure of hydride artefacts with large size and numerous tiny surface hydrides in electro polished thin-foil [10]. Those hydride artefacts were indeed induced by electro polishing.

**Figure 5:**
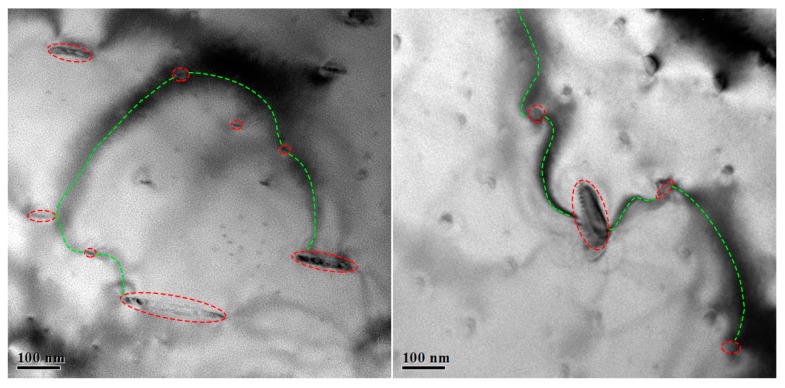
BF-TEM images of Zircaloy-4 specimens prepared by mechanical grinding, showing the intergrowth structure of hydride artefacts (red circular) and bend contours (green line).

As an added bonus, numerous bend contours could be observed in the bright-field images of specimen prepared by mechanical grinding, as shown in Figure 5. In bright-field images, the bend contours are curved black lines, which are indeed bad for observation. The bend contours seem to start at one hydride artefact and end at another. They turn to be line to connect the hydride artefacts in series. It can be observed that the bend contours are accompanied by the induced hydride artefacts, especially the hydride artefacts with large size. In TEM samples prepared by chemical polishing, the bend contours are suppressed with the reduction of hydride artefacts with large size, shown in Figure 4b. Hanlon reported similar structure of hydride artefacts induced by electron polishing [6]. They proposed that those contours were strain fields around artefact hydride. Indeed, the hydrogen is forced into the sample surface at such low temperature [6]. We need to consider the driving force for zirconium hydrogenation at low temperature. Here, we demonstrate that it is local stress that provides driving force for hydride artefact formation. As mentioned above, the hydrogen would redistribute as a consequence of stress gradients [5,13,14]. Based on this, the induced stress seems particularly important to the formation of hydride artefacts. XRD patterns indicate that stresses are indeed induced by mechanical grinding. The induced stress forces more hydrogen into samples to form hydride artefacts. That is the reason why more hydride artefacts and bend contours are present in the sample prepared by mechanical grinding.

### 3.4. Advantages and Cautions

According to our present research, the chemical polishing in TEM sample preparation process has obvious advantages compared with traditional mechanical grinding. 

Firstly, chemical polishing is of high efficiency compared with traditional mechanical grinding. It saves a lot of time and spirits. 15 min or more may be required to prepare a disk sample for twin-jet electron polishing with traditional mechanical grinding. With chemical polishing, a score of disk samples could be prepared within an hour. 

Secondly, chemical polishing actually eliminates the induced stress and improves the surface topography.

Thirdly, chemical polishing reduces the amount of large hydride artefacts and bend contours. The microstructure of TEM sample prepared by chemical polishing is closer to original condition. 

In addition, some issues should be paid more attention. During chemical polishing, continuous stirring is required to obtain sufficient reaction. Meanwhile, strict self-protection should be performed during chemical polishing.

### 3.5. Outlooks

Chemical polishing is indeed a convenient method to prepare TEM samples of Zirconium alloys. In addition, chemical polishing applies to other metallic material more than Zirconium alloys. Rzepski [21] and Van Neste [22] demonstrate that iron-base alloys and low alloys of iron could be chemical polished with a solution essentially composed of hydrofluoric acid in hydrogen peroxide to rapidly thin TEM specimens. Aluminum and Aluminum alloys could be chemical polished by the solution of H_3_PO_4_-H_2_SO_4_-HNO_3_. Another solution of 65% H_3_PO_4_ with propylene glycol or glycerine is applied to the chemical polishing of Magnesium and its alloys. In theory, all metallic materials could be chemical polished with the appropriate solution. However, more explorations are required for individual material. More attention should be attracted in the universal application of chemical polishing in TEM specimen preparation and the exploration of appropriate solutions.

## 4. Conclusions

In this study, we present the application of chemical polishing with a solution of 10HF:45HNO_3_:45H_2_O in TEM sample preparation in zirconium alloys. The solution of 10HF:45HNO_3_:45H_2_O could thin the zirconium samples uniformly with an efficiency of 25 μm per minute, meanwhile improving the surface appearance. According to the XRD patterns, macro- and micro-stress are indeed induced by mechanical grinding. The chemical polishing could eliminate the stress induced by mechanical grinding. The bend contours are accompanied by the induced hydride artefacts, especially the hydride artefacts with large size. Chemical polishing reduces the amount of hydride artefacts and bend contours. It is proposed that the induced stress provides driving force for hydride artefact formation. Compared with traditional mechanical grinding, the advantages of chemical polishing are high efficiency, free of induced stress, less induced hydride artefacts and bend contours.
